# MedGraph: A semantic biomedical information retrieval framework using knowledge graph embedding for PubMed

**DOI:** 10.3389/fdata.2022.965619

**Published:** 2022-10-19

**Authors:** Islam Akef Ebeid

**Affiliations:** Department of Information Science, University of Arkansas at Little Rock, Little Rock, AR, United States

**Keywords:** knowledge graph, natural language processing, information retrieval, biomedical digital libraries, graph embedding

## Abstract

Here we study the semantic search and retrieval problem in biomedical digital libraries. First, we introduce MedGraph, a knowledge graph embedding-based method that provides semantic relevance retrieval and ranking for the biomedical literature indexed in PubMed. Second, we evaluate our approach using PubMed's Best Match algorithm. Moreover, we compare our method MedGraph to a traditional TF-IDF-based algorithm. Third, we use a dataset extracted from PubMed, including 30 million articles' metadata such as abstracts, author information, citation information, and extracted biological entity mentions. We pull a subset of the dataset to evaluate MedGraph using predefined queries with ground truth ranked results. To our knowledge, this technique has not been explored before in biomedical information retrieval. In addition, our results provide some evidence that semantic approaches to search and relevance in biomedical digital libraries that rely on knowledge graph modeling offer better search relevance results when compared with traditional methods in terms of objective metrics.

## 1. Introduction

### 1.1. PubMed

PubMed is the National Library of Medicine's (NLM) free authoritative database of citations and search engine of more than 30 million articles in biology, medicine, pharmacy, and life sciences and across multiple curated databases such as MEDLINE^1^. PubMed is used by more than 2.5 million users each day, serving clinicians, physicians, researchers, and students (Fiorini et al., 2018). It is worth mentioning that PubMed is a database of citations, not a database of full-text articles. About two-thirds of the articles indexed in PubMed do not provide access to full texts^2^. Instead, when a free full text is available by the publisher, published as open access, or supported by a National Institutes of Health (NIH)^3^ grant, the full article gets indexed in PubMed Central^4^, NLM's accessible repository of full-text articles. Accordingly, the PubMed search engine relies on metadata and citations instead of parsing full-text articles when providing a search experience. Articles' metadata are indexed and parsed in fields to be utilized in the search process. Metadata fields include titles, abstracts, authors, journal names, publication dates, submission dates, related Medical Subject Headings (MeSH)^5^ terms, citation and references information, funding grants, and projects.

PubMed uses an algorithm that relies on fuzzy string matching to match the query with relevant citations. For example, when a user enters in the search box an author name followed by a journal name, all the articles that author published in that journal will appear. In addition, PubMed uses the Automatic Term Mapping system (ATM) (Thirion et al., 2009). The ATM system expands the input query and finds which fields the query entered intended. The expanded query is then matched with the most relevant documents using MeSH terms, keywords, and other metadata that could be treated as an index. The most relevant articles are then retrieved using the Term Frequency-Inverse Document Frequency (TF-IDF) algorithm Jones (1972) and ranked based on date or alphabetically using either the title or the author name (Fiorini et al., 2018). Other methods include ranking by date or author information. Recently PubMed deployed its newest relevance ranking algorithm named BestMatch (Fiorini et al., 2018).

BestMatch relies on a machine learning model trained on features extracted from user search logs on PubMed in the past several years. The system has been shown to outperform TF-IDF-based ranking. However, BestMatch does not consider that the user query logs that the system has been trained on contain ambiguous queries. In addition, even though the authors evaluated the system thoroughly using an A/B testing approach with real users to evaluate the ranking quality, the algorithm did not provide solutions for the problem of understanding query intentions through semantic models. For example, a user can enter the word “cancer” in the PubMed search box, and they might mean multiple things by “cancer”. For instance, they might want an article in the journal named “Nature: Cancer”. Alternatively, they might want authors who work and publish in the field of cancer. Or, they might want all relevant articles that mention cancer or research done in the field of cancer. They might also be looking for a specific citation with a title or author name, journal, and year. Alternatively, they might be looking for several articles related to cancer. Search engines and information retrieval systems such as PubMed and Google rely on objective metrics and algorithms to rank their search results. The ranking of the search results does not necessarily reflect what the user meant by the query. They, however, reflect the most objective relevance based on the text of the input query. That is done by analyzing the frequency of the strings in the input queries in the corpus of documents. In addition, other models incorporate the citation network of the documents, such as PageRank in the case of Google (Page et al., 1999). Hence, integrating semantics in search algorithms and information retrieval systems, especially in biomedical literature searches, is crucial to move toward systems that can sort out ambiguity, understand query intentions, and aid in true knowledge discovery.

In recent years and the Web 2.0 information revolution, Semantic Web technologies have proliferated (Berners-Lee et al., 2001). Semantic web technologies aim to create an understandable and readable web by machines. The graph model was introduced to represent knowledge in web pages semantically using standards such as the Resource Descriptor Framework (Lassila and Swick, 1998). The idea was driven by earlier work in digital ontology and concept maps. Knowledge graphs were then born as a data model used to store information and data semantically. Knowledge graphs have also been extended as graph databases for data persistence as it allows for a more flexible representation of data and relationships than the relational data model (Hogan et al., 2021).

### 1.2. Contribution

To help investigate the challenges associated with semantic understanding of queries when searching the biomedical literature in PubMed, we introduce MedGraph, a knowledge graph-based search engine and information retrieval method. MedGraph relies on converting the metadata associated with PubMed into a knowledge graph. The metadata includes disambiguated author names, grant information, MeSH terms, citation information, and a dataset of extracted bio entities such as drugs, genes, proteins, and species from the text of the title and the abstract of each article in PubMed. The dataset was introduced by Xu et al. (2020), and it includes NIH project involvement for each author and each article in PubMed. In addition, it has extracted biological entities using deep learning named entity recognition technique called BioBERT (Lee et al., 2020). The dataset is available as a relational database linked using each article's unique identifier PMID. The dataset contains articles from the year 1781 until December 2020. To prove the utility of MedGraph, we extracted a small dataset of 2,696 articles and their associated metadata and citation network from the PubMed dataset (Xu et al., 2020). We then extracted the entities from the dataset and linked them semantically as a knowledge graph. We then used a knowledge graph embedding method named Node2vec (Grover and Leskovec, 2016) to extract semantic features and embed the extracted knowledge graph in a Euclidean space. We then used the node vectors to rank the articles using a cosine distance similarity measure on the learned vectors according to the input query after pooling all the vectors of related first-order neighbor nodes for each article. On the query side, first, the input query is parsed and expanded using the extracted biological entities in the original dataset as an index. The expanded query is then matched to their corresponding nodes in the knowledge graph. The matched node vectors are then averaged to vectorize each query.

Using various metrics, we evaluate the proposed method against PubMed's BestMatch algorithm as ground truth. In addition, we compare our method with a traditional TF-IDF approach (Jones, 1972; Ramos, 2003). Our results show that MedGraph performs comparably to BestMatch. In addition, it outperforms the traditional TF-IDF method providing evidence that using knowledge graph-based semantic search will benefit the biomedical and life science research community when adopted as a widely used method in literature search through digital libraries.

### 1.3. Relevant previous work

Knowledge graphs (KG) (Paulheim, 2017) have been adapted to aid search engines and recommender systems. KGs are highly efficient in those applications due to their flexibility in modeling multi-cardinal relations at the entity level. For example, Xiong et al. (2017), the authors introduced explicit semantic ranking, harnessing KG embedding. The algorithm uses graph representation learning on the metadata of articles in the online search engine named Semantic Scholar (Fricke, 2018). They use a KG embedding model to represent queries and documents as vectors in the same vector space. This work is the closest to the work we present here. The authors provided strong evidence that using KG embedding in searching academic literature improves the relevance of the returned documents drastically due to the reliance on semantics and entity matching in the process. While in Wang et al. (2017), the authors demonstrated the usefulness of KGs and semantic modeling in search engines when retrieving web pages. They used a relation extraction algorithm to construct a KG. Though they have not used graph embedding, they devised a semantic matching approach based on support vector machines.

In Montes-y Gómez et al. (2000), the authors introduced extracting a KG from the text of two documents. They then measured the similarity between these two graphs extracted from the two articles, combining relational and conceptual similarities. In Ebeid et al. (2021), the authors showed the utility of ranking methods on embedded KGs using simple cosine distance metrics to perform tasks such as link prediction in the biomedical domain. While in Matsuo et al. (2006), the authors described a system built using keyword co-occurrence matching. They remodeled the keyword matching process as a graph and applied a graph clustering technique to match keywords and queries. In Blanco and Lioma (2012), The authors modeled the text in documents as a graph instead of a Bag of Words model (BoW). Then, they used PageRank (Page et al., 1999) to derive similarity measures between documents. At the same time, the authors (Farouk et al., 2018) argued that graph modeling could enhance search relevance results based on context rather than just string similarity. They developed a system where the input documents and indices are converted to a KG. Their findings support (Ma et al., 2016), where they drove the point that graph-based search engines are highly efficient and valuable despite their challenges. Evidence of the utility of graph-based search is strengthened in Guo et al. (2021). The authors constructed a network of the standardized MeSH headings assigned to articles in MEDLINE (Motschall and Falck-Ytter, 2005). The relationships between the MeSH headings were modeled as a graph where the edges represent different hierarchical roles in the original MeSH coding system. The graph of MeSH headings was then fed to various graph embedding algorithms. The output was a learned feature vector representing each MeSH heading for each node. The data set is helpful in downstream biomedical computational tasks.

While in Wang J. Z. et al. (2014), the authors used an efficient graph-based search engine on par with PubMed. Their approach tackled the problem of returning relevant documents from three angles. They first built a parallel document indexer. Second, they modeled each article's metadata, such as MeSH terms and keywords, as a graph and applied a personalized PageRank (Lofgren et al., 2016) to rank the concepts in the built graph, followed by TF-IDF (Pita et al., 2018) to rank the documents relative to a query. Third, they included the user's search behavior as a factor in relevance, similar to BestMatch (Fiorini et al., 2018). Despite its efficiency compared to PubMed, the algorithm requires user input and is not fully unsupervised. The BestMatch (Fiorini et al., 2018) is the newest algorithm used by the PubMed search engine to find the most relevant articles to a user's query. BestMatch relies on extracting features from articles and including prior user search logs into a relevance ranking prediction model. The model then finds the most relevant results personalized to each user. BestMatch provides excellent results compared with previous approaches in PubMed, yet it does not consider any semantics failing to distinguish ambiguity in queries.

In the next section, we describe our methodology and framework proposed in this article. In Section 3, we describe our evaluation experiments and results. Section 4 discusses the results, implications, and future work. We conclude in Section 5. A complete bibliography is available in Section 6. An additional literature review is included in the Supplementary material.

## 2. Method

**Figure 1:**
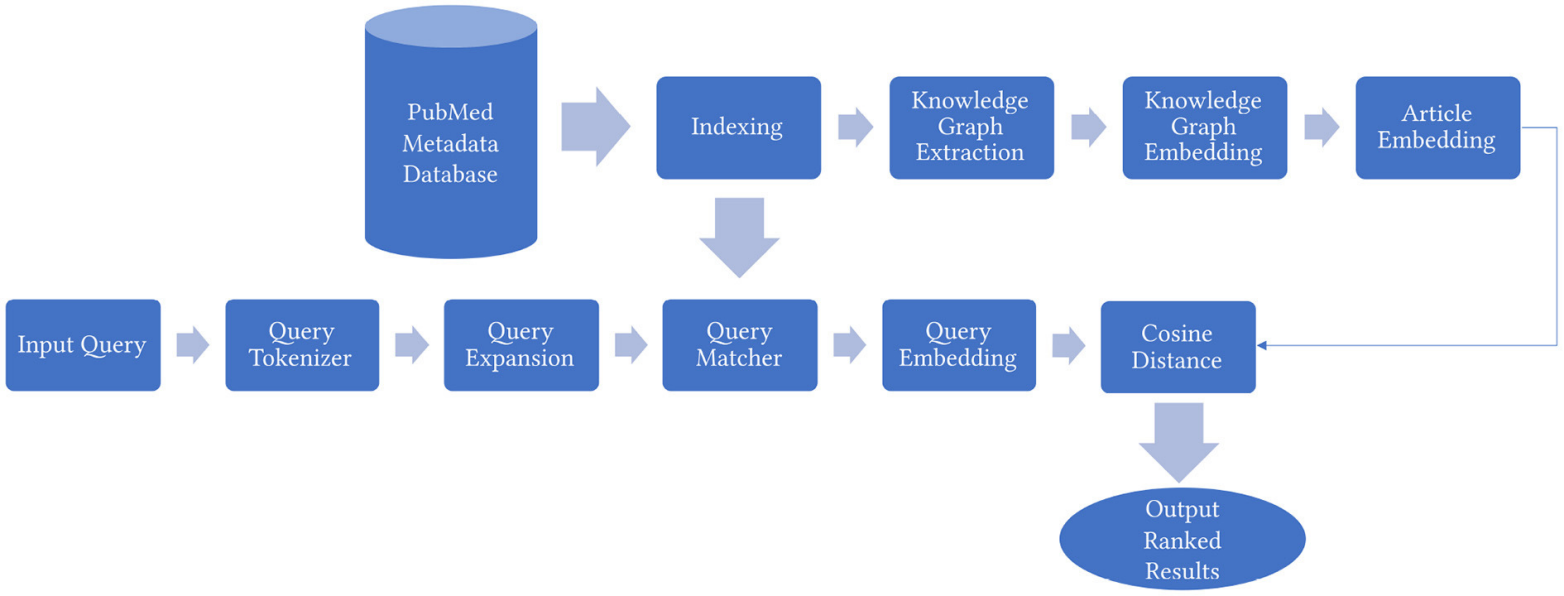
An overview of the MedGraph framework.

In this section, we explain in detail the proposed KG based biomedical information retrieval framework MedGraph as shown in Figure 1. An additional illustrative example of our framework's pipeline is available in Supplementary Figure 1.

### 2.1. The PubMed metadata database

In Xu et al. (2020), the authors extracted a metadata database from the corpus of the PubMed articles available from 1781 until December 2020 (30 million). The extracted information includes names of biological entities such as genes, proteins, species, drugs, and diseases and disambiguated author information and citation information. The primary purpose of that dataset was to create a full KG of the articles in PubMed. The extracted biomedical KG could be used in various biomedical information retrieval and data mining tasks. Here we utilize the extracted biomedical knowledge graph described in Xu et al. (2020). The dataset comes as a relational database linked by a unique identifier, each article's identifier in PubMed, also known as the PMID. Those account for 31, 929, 000 articles. Author information from each article, including first names, middle names, last names, and affiliations, has been extracted and disambiguated in separate tables. In addition, the disambiguated authors have a unique identifier of AIDs.

Table 1 provides statistics and a description of the PubMed relational database for essential tables. The original dataset contains 27 tables linked by PMID. Here we extract metadata from seven tables. In addition, we do not use 31 million articles for our dataset. Instead, we choose a subset of articles that have been submitted to journals between the dates of 2/1/2019 and 2/3/2019. This subset of the articles yielded 2,696 articles when queried on PubMed. We then use the 2,696 articles to extract a first-order citation network from the table *C04_ReferenceList*. The citation network produced 100,456 articles. Finally, for the 100,456 articles, we extracted the rest of the metadata from the tables listed above, which will be described later.

**Table 1:** A description of main tables in the downloaded PubMed dataset provided in Xu et al. (2020).

**Table**	**No. of rows**	**No. of distinct entities**	**Description**
A01_Articles	31,928,777	31,926,861	A table containing PubMed articles' bibliographic information.
A02_AuthorList	131,446,038	18,519,492	A table containing PubMed authors and their unique identifiers.
B10_BERN_Main	295,921,671	20,136,150	A table containing all types of extracted bio-entities by BioBERT are used in both building the Knowledge Graph and as an index.
C03_Affiliation_Merge	62,015,712	9,502,394	A table containing affiliations and extracted fine-grained items.
C05_NIH_PubMed	22,946,601	116,530	A table containing projects from NIH ExPORTER and mapping relation between PI_ID, PMID, and AND_ID.
C04_ReferenceList	633,401,975	23,856,949	A table containing reference relations between PMID and reference PMID. It was extracted from the Web of Sciences.

### 2.2. Indexing

Indexing is simply mapping unique vocab to documents or the opposite like the index at the back of a book. You can expand that definition and match the extracted unique vocab to a dictionary (Xu et al., 2020). The index here is the mapping between the limited unique vocab of the recognized entities and their respective documents which is enough for our task. The difference between our indexing strategy and a more generalized approach is that we did not expand the index to include all unique entities we just limited the index to the extracted biomedical terms. In addition in our case, we use the terms extracted during the named entity recognition as a limited index. Moreover, the table named *B10_BERN_Main* represents the names of drugs, genes, diseases, and species extracted using named entity recognition using the biomedical deep learning language model BioBERT (Lee et al., 2020) in the dataset presented in Xu et al. (2020), which acts as an index in addition to being part of the KG that we will describe its extraction later in the following subsection. In addition, the index will be used to match input user queries and expansion and create query vectors. More formally, each article *p* ∈ *P* will contain a set of biological entity mentions *m* ∈ *M*. Each mention is part of a set of mentions that distinguish each unique biological entity *b* ∈ *B* where *M′* ⊆ *M* and *b* → |*M′*|. In addition, each unique biological entity has a type that can be one of four types[ drug, disease, gene, species] where *b*(*t*) ∈ *B*(*T*) *and T* = [*drug, disease, gene, species*]. Hence the relationship becomes *p*[*b*(*t*)] ∈ *P*[*B*(*T*)] ∀ *b* → |*M′*|. Note that we only use extracted biological entities from the text of each article to index our corpus of articles instead of using MeSH terms or UMLS (Bodenreider, 2004) vocabulary, which is considered a standard approach in work that has been done before in biomedical information retrieval and text mining.

### 2.3. Knowledge graph extraction

**Figure 2:**
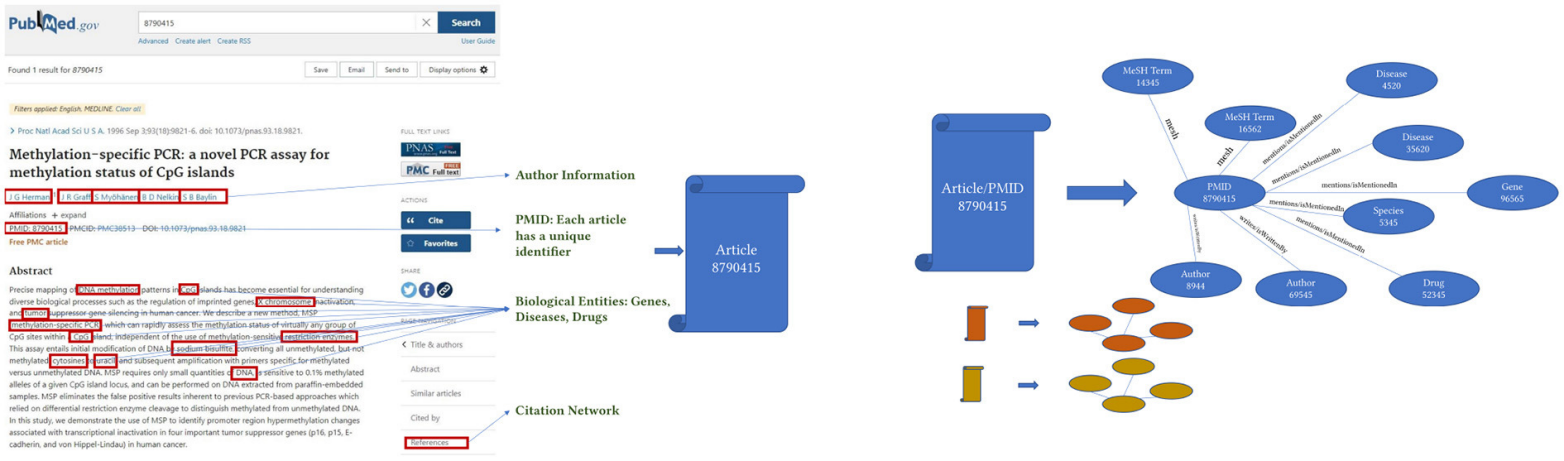
The image represents how each article is converted to a concept graph or a smaller knowledge graph.

KG extraction converts the relational database of the PubMed metadata to a graph of interconnected entities, as shown in Figure 2. For each article, we first extract all author names, names of drugs, genes, proteins, diseases, and species, and related MeSH terms and Chemical Substances terms from the tables described above. Then, the unique identifiers representing each entity create the KG. As described before, KGs are represented as a list of triples. For example, in our case, when we extract an author name for an article from the metadata database, we represent that information as [“article/pmid/86509”, “isWrittenBy/wrote”, “author/aid/6754”]. Similarly, when we extract a drug name from an article, that information is represented as [“bioentity/drug/1256”, “isMentionedIn/mentions”, “article/pmid/78456”]. In addition, if an NIH grant or project funded an article, that information will be represented as [“ article/pmid/5678”, “isFundedBy/funds”, “nih_project/project_id/4123”]. Note that the relationships are represented equally as the data in this KG model compared with a relational model.

**Figure 3:**
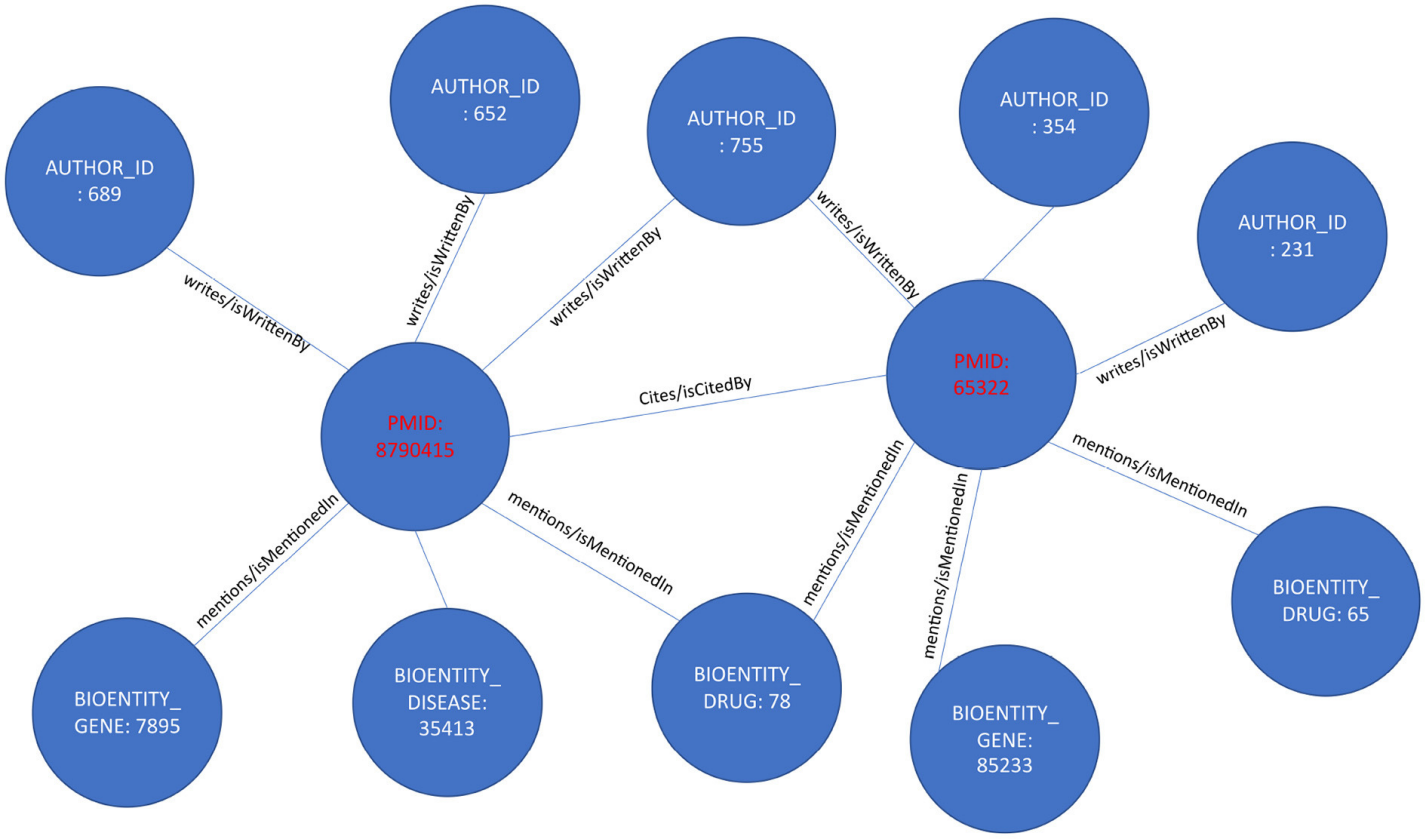
A part of the extracted knowledge graph.

Accordingly, each article and associated metadata will be represented as a mini KG or a concept graph, as shown in Figure 2. Those mini KGs or concept graphs could be seen as subgraphs of a larger encompassing KG. In our case, we link all the subgraphs in two ways. First, we use the citation network provided in table *C04_ReferenceList*, representing extracted citation information from PubMed and Web of Science. The citation network provides the edges necessary to link most articles using the relationship “isCitedBy/cites.” For example, two articles will be linked and represented in the knowledge graph as a triple [“article/pmid/652148,” “isCitedBy/cites,” “article/pmid/415923”]. Second, since the authors and the names of drugs, diseases, genes, and proteins are disambiguated and unique, if an author appears with multiple names across several articles, all the names they appeared with will have the same author identifier number. Similarly, they will have the same unique identifier if they occur with different names, such as Aspirin and NSAID for drugs, proteins, genes, and species. Moreover, we create a mini KG for each article using a unique identifier. The linked KG will also be semantically related because an author will appear in multiple articles, a drug name in various articles, and the citation network connects all articles. The final KG will be a semantically linked network representing articles, authors, NIH grants, drugs, diseases, and genes. Extracting a KG dataset as described above for the whole corpus of articles in PubMed is a daunting task. We extract only a small subset of articles with their citation information to prove the concept. KG extraction can be formalized by seeing each subject and object in the extracted triples [*v*_*i*_, *r*_*k*_, *v*_*j*_] as nodes *v* of type *l* in a KG *v*(*l*) ∈ *V*(*L*) where each node has a type *l* ∈ *L* where *L* = [“article”, “author”, “gene/protein”, “drug”, “disease”, “species”, “nih project”, “mesh term”, “chemical substance”]. Edges in the KG are equivalent to verbs or predicates in the triple representation, as shown in Figure 3. Each edge *e*(*k*) ∈ *E*(*K*) has a type *k* ∈ *K* where *K* = [“isCitedBy/cites”, “isMentionedIn/mentions”, “isFundedBy/funds”, “mesh”, “isRelatedTo/relates”]. Hence the triple relationship can be reformalized to *G* = (*V, E*).

Regarding the validity of the extracted KG please refer to Xu et al. (2020). As mentioned before the authors extracted entities using BioBERT, a finely tuned state-of-the-art biomedical BERT model. The validation was done by comparing the results to a pretrained general BERT model on the general domain corpus. The relations were validated using multiple normalization models and dictionaries such as GNormPlus for Gene/Protein and Sieve-based entity linking for Diseases. Author disambiguation was validated using the NIH ExPORTER and NIH-funded research databases.

### 2.4. Knowledge graph embedding

**Figure 4:**
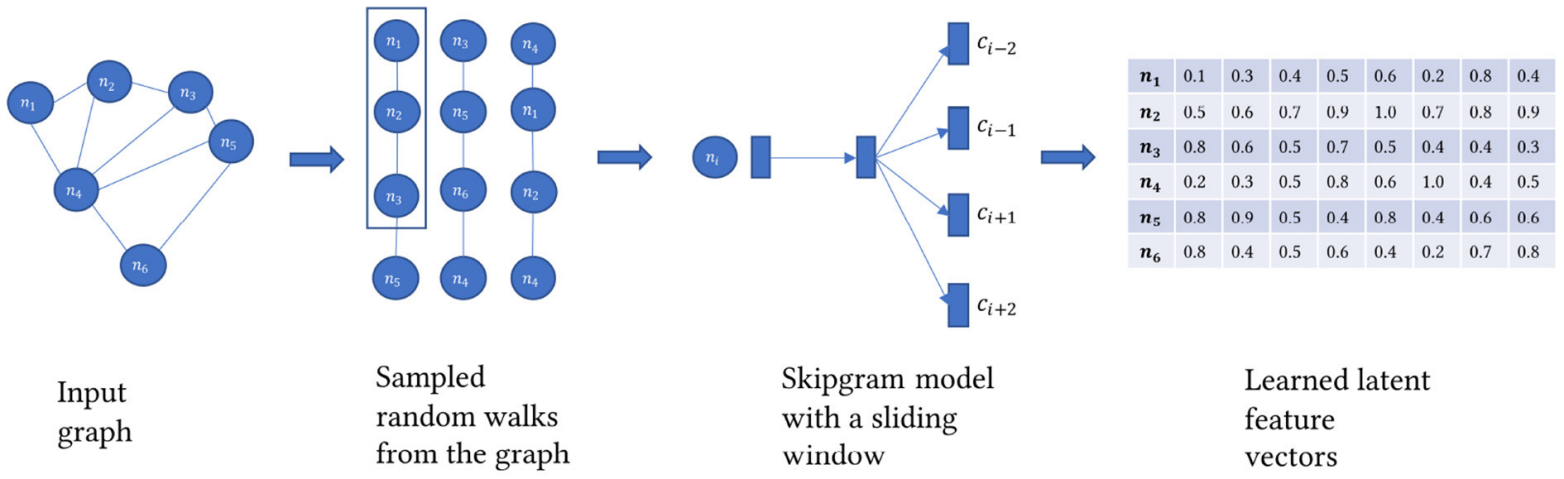
A representation of the Skip-gram model.

Knowledge graph embedding models can be transductive as in learned from the structure of the graph itself (Perozzi et al., 2014; Tang et al., 2015; Grover and Leskovec, 2016). Or they can be distance based by forcing a scoring function to evaluate the plausibility of the triples in the KG (Bordes et al., 2013; Lin et al., 2015). Or based on end-to-end graph-based deep learning models such as Graph Neural Networks (Kipf and Welling, 2016). More knowledge about graph embedding can be found in Wang et al. (2017). Here we aim to learn a set of feature vectors for each node or entity in the KG as shown in Figure 4. The feature vector needs to encode the structure of the graph. More formally, for the graph *G* = (*V, E*) a matrix *X* ∈ ℝ^*d*^ is learned *via* the function *f*:*v ϵ V* → ℝ^*d*^. One of the constraints on the learned embedding matrix is that it can be decomposed to $X\;=\;Z_{v}^{T}Z_{u}$ so that *X* preserves the similarity between its component matrices where *v* ∈ *V* and *u* ∈ *V* and $Z_{v}\equiv X^{T}$ and *Z*_*u*_≡*X*. Preserving the similarity is learned through predicting the probabilities of co-occurrence between 2 nodes in the same neighborhood within a specific context window *C* after sampling the graph using a random walk strategy to a size of a corpus sampled nodes, *T*.


(1) $$\begin{array}{l} P\left(v_{1},v_{2},v_{3},\;\ldots ,\;v_{t}\right)=\;\frac{1}{T}\;\overset{t=1}{\underset{T}{\sum }}\underset{-c\;\leq \;j\;\leq \;c,\;j=0}{\sum }\log P\left(v_{t+j}|v_{t}\right) \end{array}$$

Where *c* ∈ *C* and *t* ∈ *T*. *v*_1_, *v*_2_, *v*_3_, …, *v*_*t*_ are sampled from the first order neighborhood *N* of a randomly chosen node *v*_*i*_. To train matrix *X*, we approximate the probability *P*(*v*_1_, *v*_2_, *v*_3_, …, *v*_*t*_) over positively and negatively sampled and labeled nodes using a sliding window on the sampled chains of nodes from the graph as described in equation 1. Nodes within the context window are labeled 1, while nodes outside the context window are labeled 0. A sigmoid function is then used to normalize the parameters of the matrix *X*. A backpropagation phase then takes place to optimize the loss function:


(2) $$\begin{array}{l} J_{t}\left(\theta \right)\;=\;\log\sigma \left(u_{0}^{T}v_{c}\right)\;+\;\underset{j\;=\;P\left(V\right)}{\sum }\log\sigma \left({-u}_{j}^{T}v_{c}\right) \end{array}$$

Where *u* and *vϵ V* and *u*_*i*_ and *v*_*i*_ are row vectors ϵ* X*. The previously described algorithm is the Skip-gram model introduced in Mikolov et al. (2013). It is worth mentioning that first-order neighborhood means one edge at a time. It is different than the walk length. Other types of graph embedding algorithms might take into consideration 2nd and 3rd order. But in general, it is computationally impractical and intractable to take more than that. To extract KG embedding representations, we use Node2vec, the algorithm described in Grover and Leskovec (2016). Node2vec performs a modified version of the random walk strategy in Perozzi et al. (2014), including parameters *p* and *q* to control the sampling strategy. The *p* parameter controls the likelihood of the walk revisiting a node. The *q* parameter controls whether the search is constrained locally or globally. Given *q*>1 and a random walk on an initial node, the random walk samples nodes closer to the initial node as in Breadth-First Search. Whereas, *q* < 1, random walk samples nodes further from the initial node like a Depth First Search. This customizability in search behavior allows the random walker to capture diverse structural and topological properties within the graph. The sampling strategy builds a corpus of walks starting from each node. The Skip-gram model trains on this corpus to generate a unique embedding vector for each node in the KG. Once the model finishes training, we get an embedding vector of size d for each node regardless of its type, whether an article, author, drug, disease, gene, NIH project, or MeSH term.

### 2.5. Article embedding

Our goal is to build a backend KG-based embedding model used by a front-end search engine to rank articles relevant to specific user queries. This step uses a pooling operation averaging all the node embedding vectors of all types of nodes connected to each article node in its first-order neighborhood. We created the article embedding model in two stages. First, we performed the pooling operation of averaging all the nodes of the articles as described before mentioned in the citation network, which gives us 100,456 articles. Next, we did a second pooling operation where we averaged the first-order neighbors of articles for the 2,696 articles we intend to search.

**Figure 5:**
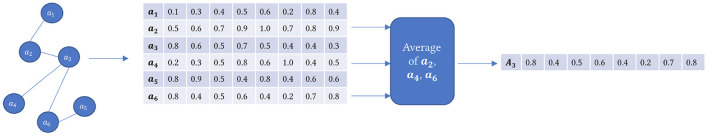
How article embeddings are generated.

In Figure 5, the graph on the left is our KG, where we only have article nodes along with other node types as shown in Figure 3. For example, suppose we want to calculate the embedding for article *a*3, one of the 2,696 articles, but it is also connected to other article nodes in the graph. So we average all the embedding vectors of the neighboring articles only, that is, *a*2, *a*4, and *a*6, and the resultant vector will be the one representing *a*3.

### 2.6. Query tokenizer

This module acts as an interface with the user. It takes user queries and parses them. The input queries are assumed to be in English and are tokenized by splitting over white spaces after removing punctuation, stop words, and verbs. For example, a query like “show me articles on depression and type 2 diabetes” after tokenization it will be reduced to [“articles,” “depression,” “type,” “2,” “diabetes”]. The output keywords will be passed to the query expansion module. Note that the assumption here is that the query should include keywords in the index.

### 2.7. Query matcher

The list of extracted keywords is then expanded using a sliding window of sizes 2, 3, and 4. The sliding window's function captures multiple tokens from the initial keyword list. It slides over the list of keywords and expands it. For example, our list of keywords [“articles,” “depression,” “type,” “2,” “diabetes”] will be expanded to [“articles,” “depression,” “type,” “2,” “diabetes,” “articles depression,” “depression type,” “type 2,” “2 diabetes,” “articles depression type,” “depression type 2,” “type 2 diabetes”]. The expanded list of keywords is then matched using a Levenshtein string distance comparator to the index. The index contains all the extracted biological entities from the articles and their unique identifiers and locations. For the matched mentions in each article in the index, each biological entity's unique identifier will be extracted and passed to the next step. Similar to PubMed the system exits if the keywords are not found in the index.

### 2.8. Query embedding

This step aims to find all the nodes in the KG with the same identifiers as the identifiers returned by the query matcher. After identifying the nodes, their corresponding learned embedding vectors from the KG embedding step is extracted. All the vectors are averaged to a single vector in a pooling operation like Figure 5. The single vector becomes our query embedding vector.

### 2.9. Cosine distance and ranked results

In a Euclidean space, the cosine of angle θ between two vectors *A* and *B* is determined using the relationship:


(3) $$\begin{array}{l} similarity\;=\cos\left(\theta \right)=\frac{AB}{\left||A||||B|\right|} \end{array}$$

Since our KG has been embedded in Euclidean space, the similarity between two nodes is equivalent to the cosine of the angle between the two vectors representing the two nodes. So at this point, we have a query vector and a set of article vectors. A simple operation between the query vector and the article vectors would yield the list of articles relevant to the query vector. When sorted by the cosine score, the list of articles will be presented as ranked retrieved articles.

## 3. Evaluation

### 3.1. Dataset

For general tasks in information retrieval there exists multiple benchmark datasets (Thakur et al., 2021). However, in biomedical information retrieval, there is a lack of benchmark datasets specific to this particular task (Fiorini et al., 2018). That said it is not unusual in information retrieval for researchers to device their evaluation procedure and dataset like we did here. The only difference is that here we did not perform A/B testing with users. We used the common heuristic mean average precision (MAP) on a reproducible dataset. The contribution of this work lies in the fact that an information retrieval researcher can take this tested framework and apply it to another biomedical digital library or further test it on any digital library. Hence we extracted a proof of concept dataset from the PubMed database described in Xu et al. (2020) and available at http://er.tacc.utexas.edu/datasets/ped. The database contains 3, 190, 000 articles indexed from the year 1781 to December 2020. We extracted our target dataset of 2,696 articles submitted to journals between 02/01/2019 and 02/03/2019. We came about those dates by examining the number of articles that have been submitted to journals for each month in the past 5 years in PubMed. We then chose the month with the least number of articles submitted, February 2019. Still, the dataset at that point was too large. Note that we include extracted articles, but we also query the reference table to extract the first order citations of each article, so the number grows exponentially. Accordingly, we kept reducing the number of days where articles were submitted to their journals until we got a reasonable size dataset. The dataset was extracted by first querying the PubMed online search engine^6^ for the articles that were submitted to their journals each month for each year since 2019:

((((”2019/*month*/01”[*Date*−*Completion*]:”2019/*month*/30”[*Date*−*Completion*]))))

Then the month with the least number of completed and submitted articles was chosen across all years. Then we adjusted to choose only 3 days since the size of the yielded citation network would have been beyond the scope of this study. We then settled for the dates mentioned above and queried PubMed with the query, which yielded 2,696 articles:

((((”2019/02/01”[*Date*−*Completion*]:”2019/02/03”[*Date*−*Completion*]))))

We then extracted the PMIDs of those articles. The extracted PMIDs were used to query the downloaded PubMed database to extract all the necessary metadata for each article. We first extracted the citation network of the 2,696 articles, which yielded 100,456 articles, including the 2,696 articles. For the 100,456 articles, all the metadata has been extracted, including author information, MeSH terms, Substances, NIH project involvement, extracted drug, disease, and protein names, and citation network from Table 1. The extracted metadata was used to create the KG as described in Figure 2. The final KG is a multi-undirected graph with the following description in Table 2. The total nodes in the graph were 578, 453, representing nine types of entities; authors, articles, NIH projects, MeSH terms, registered chemical substances, diseases, drugs, genes, and species. Most of those nodes were author nodes, followed by article nodes, then several NIH projects, MeSH term nodes, and extracted biological entities. Note that what defines a node in a graph is its identifier. Each node in the KG is identified by its original identifier concatenated to its type with a slash. For authors, identifiers are Author IDs (AIDs) in the database, PMIDs identify articles, Project IDs identify NIH projects, Header IDs identify MeSH terms, and extracted biological entities are identified by their unique Entity ID assigned by BioBERT in the original paper (Xu et al., 2020). For example, an article node will appear in the KG “*article*/*pmid*/652148.” On the other hand, edges in the KG are identified by their edge type. Here we identify nine relationships represented with edge labels, as shown in Table 2.

**Table 2:** The description of node and edge types in the extracted knowledge graph.

**Node/Edge type**	**Count**
No. of nodes	578,453
No. of author	393,864
No. of article	100,456
No. of NIH projects	27,109
No. of MeSH terms	20,015
No. of chemical substances	9,686
No. of disease	9,594
No. of drug	8,762
No. of gene	6,094
No. of species	2,873
No. of edges	2,226,999
No. of article-relatedTo-MeSHTerm	1,049,789
No. of article-writtenBy-author	596,340
No. of article-mentions-disease	176,516
No. of article-mentions-drug	108,435
No. of article-cites-article	104,138
No. of article-mentions-species	70,694
No. of article-mentions-gene	56,337
No. of article-isFundedBy-NIHProject	54,751
No. of article-relatedTo-substances	9,999

### 3.2. Experimental setup

We then trained the resultant KG to extract node embedding vectors using a Node2vec (Grover and Leskovec, 2016) approach implemented using Python 3.8 and the library Stellargraph (Data61, 2018). The algorithm first runs a biased random walk sampling algorithm on the graph to sample chains of nodes using the breadth-first bias parameter *q* = 0.5 and the depth-first bias parameter *p* = 2 with a walk length of 50 and 5 walks per node. The sampled corpus of node walks is then used to train a Skip-gram model as described in Figure 4. Next, we tuned the Skipgam model over multiple iterations to yield the best MAP value. The final model was trained using the vector size 128 chosen from a list of [12, 24, 48, 64, 128, 256], context window size 5 chosen from the values [3, 5, 7, 12] which are mostly commonly used in the literature, and the number of negative samples was 7 from the values [7, 10, 20] also from the most commonly used values in the literature. The model was trained on a Windows PC with an Intel *i*7 processor and 32 GB of RAM. We also implemented and trained a TF-IDF model on our corpus of 100,456 articles and then extracted the TF-IDF vectors for the 2,696 target articles to compare against our method. With the help of the Python library Gensim (Rehurek and Sojka, 2011), we first extracted a dictionary of unique tokens in the corpus and then trained a Bag of Words model. The Bag of Words model was then used to train the TF-IDF model, yielding a vectorized document matrix and unique vocabulary. We evaluated MedGraph to assess the quality of our KG embedding based on relevance ranking against PubMed's BestMatch algorithm as ground truth. We extracted a set of 15 queries from PubMed, and we applied the search to the articles that were completed between the dates of 2/1/2019 and 2/3/2019. The 15 queries were chosen randomly from the extracted index of biological entities as described in Section 2.2. They contained the names of diseases and drugs, as shown in Table 3. For example, for the query “type 2 diabetes,” we use the following query to search PubMed and then download the resultant PMIDs of the ranked articles.

**Table 3:** A description of the queries we used to evaluate the system against PubMed's BestMatch ranked results were used as a ground truth.

**Query ID**	**Text**	**No. of relevant documents**	**No. of tokens**
1	Alcohol	37	1
2	Amino acids	11	2
3	Bacterial infections	6	2
4	Basal cell carcinoma	3	3
5	Bipolar disorder	10	2
6	Cancer	320	1
7	Diabetes	59	1
8	Hepatitis c virus	3	3
9	Histamine	2	1
10	Insulin	25	1
11	Loss of muscle strength	1	4
12	Pediatric cancer	1	2
13	Trauma	22	1
14	Type 2 diabetes	22	3
15	Urinary tract infection	5	3

((((”2019/02/01”[*Date*−*Completion*]:”2019/02/03”[*Date*−*Completion*])))) *AND*(*type*2*diabetes*[*TextWord*])

Then for each query, we rank the articles based on the cosine distance metric by comparing the query vector to the article vectors described in Figure 1. We then prune the list of the resultant ranked retrieved articles by *K*. That means we choose the top *K* elements of the ranked retrieved articles from MedGraph. Then we compute the number of relevant articles, the number of retrieved articles, and the number of relevant articles retrieved. We then compute precision, recall, and F1-Score. Precision is the number of relevant articles retrieved over the total number of relevant articles. The recall is the number of relevant articles retrieved over the total number of retrieved articles. Moreover, the F1-Score is the harmonic mean of precision and recall.

We also compute the Mean Average Precision (MAP) across queries (Aslam and Yilmaz, 2006). MAP is a widely used metric in information retrieval to evaluate search engines. It focuses on precision since recall can be misleading in some cases. To compute MAP, we first calculate the average precision for each query. That is done by finding each retrieved article in the ground truth and for top *K*. Then computing precision at each article in the retrieved articles. That is followed by averaging the precision values across all retrieved articles *K*. Then averaging across all the queries.

## 4. Results

**Figure 6:**
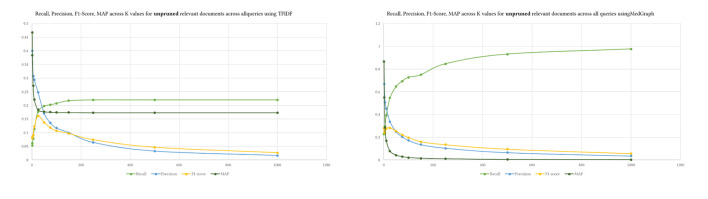
MedGraph vs. TFIDF on all four metrics.

Table 4 presents the results of the four metrics we described in the previous section. We ran 12 levels of *K* for both our method MedGraph and the standard TF-IDF (Ramos, 2003) approach for ranking relevant documents. Our results indicate that MedGraph has outperformed TF-IDF on the PubMed BestMatch dataset at various levels of *K* and across all queries and metrics. The only exception is that MAP at higher *K* levels was higher for TF-IDF. That might explain why TF-IDF returns more relevant documents but does not rank them higher, while MedGraph might retrieve less relevant documents more semantically related and ranked closely. In addition, both precision and recall for MedGraph were consistently higher. The recall increased exponentially with higher *K*, and precision decreased exponentially with higher *K* levels, as demonstrated in Figure 6.

**Table 4:** Results averaged across the 15 queries on different K levels.

**Metric**	**Method**	**K = 1**	**K = 2**	**K = 5**	**K = 10**	**K = 25**	**K = 50**	**K = 75**	**K = 100**	**K = 150**	**K = 250**	**K = 500**	**K = 1,000**
Recall	TFIDF	0.053	0.062	0.078	0.113	0.177	0.197	0.202	0.207	0.217	0.22	0.22	0.22
	MedGraph	**0.227**	**0.245**	**0.297**	**0.392**	**0.545**	**0.646**	**0.693**	**0.726**	**0.749**	**0.846**	**0.931**	**0.976**
Precision	TFIDF	0.467	0.4	0.307	0.293	0.248	0.171	0.136	0.117	0.1	0.064	0.032	0.016
	MedGraph	**0.867**	**0.667**	**0.507**	**0.453**	**0.336**	**0.248**	**0.202**	**0.172**	**0.134**	**0.103**	**0.064**	**0.034**
F1-Score	TFIDF	0.081	0.083	0.09	0.122	0.161	0.138	0.118	0.107	0.097	0.074	0.046	0.026
	MedGraph	**0.279**	**0.245**	**0.235**	**0.276**	**0.282**	**0.253**	**0.221**	**0.197**	**0.161**	**0.134**	**0.095**	**0.056**
MAP	TFIDF	0.467	0.383	0.272	0.221	0.184	0.177	0.175	0.174	0.174	0.173	0.173	0.173
	MedGraph	**0.867**	**0.55**	**0.284**	0.168	0.077	0.041	0.028	0.021	0.014	0.009	0.004	0.002

Bold values indicate the instances where MedGraph has outperformed TF-IDF on different metrics.

MedGraph had higher MAP and F1-Scores across all *K* levels due to its higher recall and precision. The highest difference between MedGraph and TF-IDF was at *K* = 1, indicating that the first document in the retrieved documents almost always existed in the ground truth dataset. However, recall was the lowest because most of the relevant documents did not exist in the first position in the retrieved documents. Of course, as we increase *K*, the recall increases, indicating that most of the relevant documents in the ground truth appeared in the retrieved documents. At *K* = 10, MedGraph started underperforming on MAP while TF-IDF stayed consistent at higher *K* levels. That is because MedGraph ranks a small number of the relevant documents highly, while many of the documents do not appear in MedGraph. The documents that appear in the retrieved documents are ranked closely and higher due to the semantic nature of the algorithm, while the documents that are not closely ranked and in the top are usually ranked lower and tend to be spread out.

Alternatively, in other words, MedGraph produces relevant articles that are closely ranked together due to the semantic nature of the algorithm. In contrast, TF-IDF has almost the same number of relevant articles but is not ranked closer together. Finally, we computed the four metrics by pruning the top *K* ground truth results from relevant documents from BestMatch.

**Figure 7:**
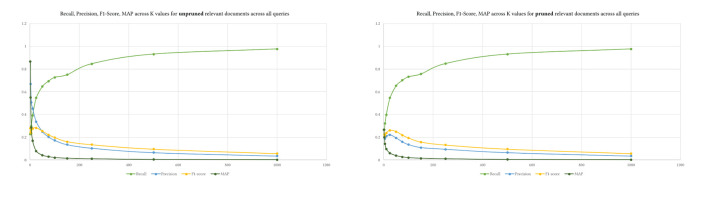
The difference between pruned and unpruned retrieved results.

We used the same *K* levels provided to prune the retrieved and relevant results. Figure 7 shows the difference between pruning the relevant ground truth articles and not pruning them. The values of recall and F1-Score do not differ between both approaches. Yet, precision and MAP are higher when the relevant documents are not pruned using *K*. Pruning perhaps provides a mechanism to control the ground truth dataset. We do not know how exactly BestMatch ranked it. The returned BestMatch articles from PubMed have different retrieved articles without explanation, as shown in Table 4. Hence pruning might make sense in some cases depending on the evaluation dataset.

**Figure 8:**
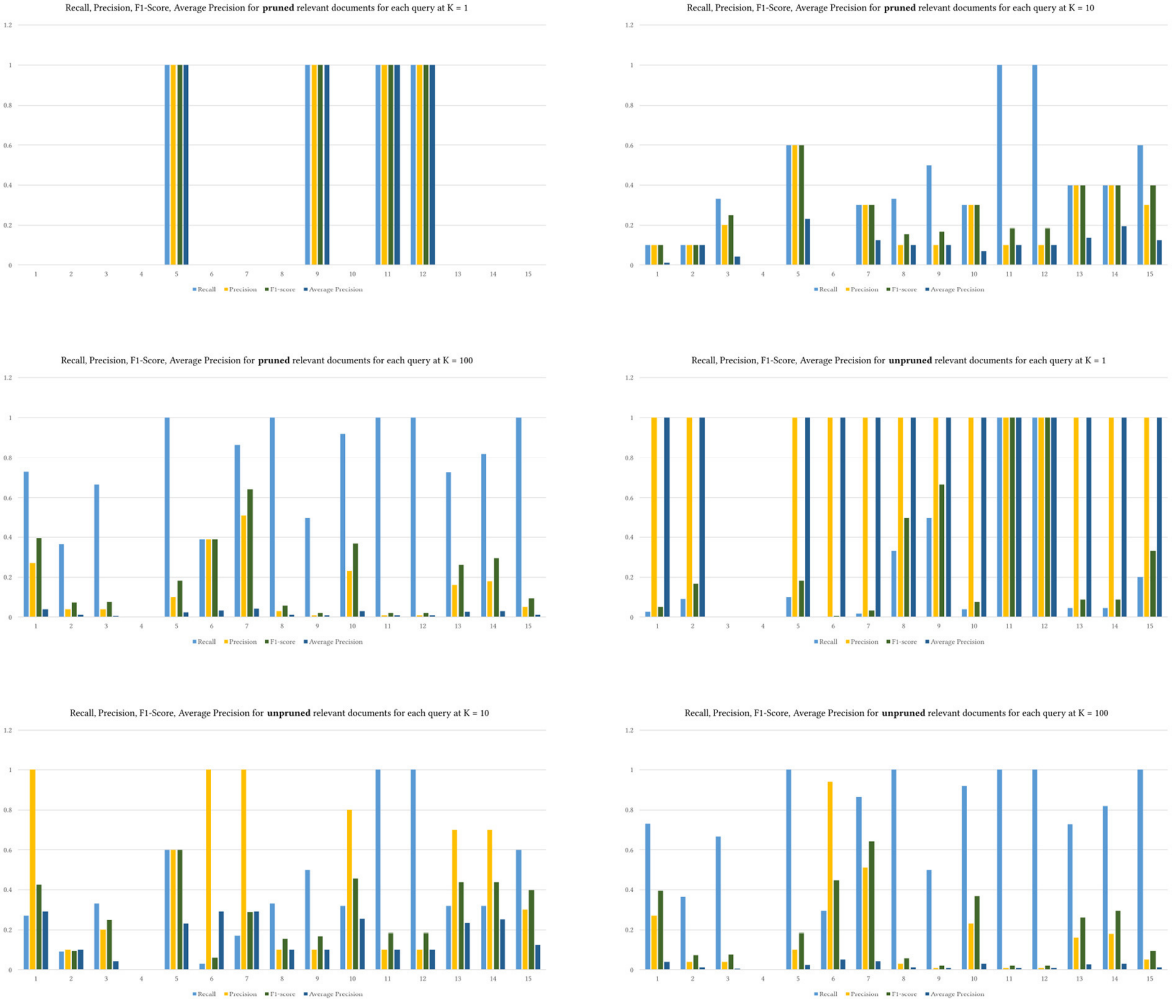
The four metrics across various levels of K over the 15 queries. Upper is pruned, and lower is unpruned relevant documents.

That is also seen in Figure 8, where precision was much higher across queries with unpruned relevant results, indicating that MedGraph retrieved almost all of the relevant results compared with BestMatch.

## 5. Discussion

This work provided evidence of the utility and efficiency of KG-based methods in information retrieval, especially in the biomedical field. We highlighted the need for more techniques that rely on semantic understanding of queries and datasets to aid in automated knowledge discovery and information organization. KGs have been around for a while, yet they have not been fully utilized in search engines. Approaches such as BestMatch for PubMed are very efficient but do not understand semantics and are trained on user query logs that might change over time, requiring retraining. Traditional TF-IDF approaches do not rely on semantics and are almost outperformed by newly developed methods like ours. The results also indicated that MAP alone is not enough as an evaluation metric. The ranking is usually evaluated using A/B testing approaches involving user studies and metrics that would include users ranking relevance by hand and then computing metrics such as Normalized Discounted Cumulative Gain (Busa-Fekete et al., 2012). Precision as a metric is very informative in evaluating how many relevant articles were retrieved and, in our case, MedGraph. It highlighted its superiority. Nevertheless, metrics such as recall can be misleading. For example, if the system only retrieved one document, but that document is in the relevant documents no matter the rank, then recall shall be 100%. Precision acts as a self-assessment of the retrieved articles by MedGraph because it compares the numbers of retrieved relevant articles to the number of retrieved articles regardless of the number of relevant articles.

In our future work, we plan to conduct a user study where each user, typically a biomedical researcher or a medical student, will be invited and asked to rank documents based on specific queries. We will create our ground-truth dataset instead of relying on BestMatch as our ground truth. We also plan to expand the scope to extract a KG from the entire dataset of 30 million articles (Xu et al., 2020) and compare our model with BestMatch and TF-IDF using our ground truth. Node2vec represents a basic model incapable of encoding heterogeneity in KGs. Heterogeneity refers to a KG having more than one type of node and more than one type of edge or relationship. Hence more sophisticated embedding algorithms such as Wang Z. et al. (2014), which focuses on embedding not just the structure but also the relations in the KG could be used. In addition heterogeneous graph neural networks (Wu et al., 2020) could be also used and both might provide better results. In that light, we plan to experiment with various other KG embedding models (Wang et al., 2017) like GraphSAGE that are capable of handling dynamic KGs. In addition we will experiment with embedding models capable of capturing more semantics in the training of node embeddings, expanding our query matching capabilities to include more than four tokens, and handling out-of-context queries. Moreover, we plan to have even more metadata nodes in our KG with the potential of enriching the KG with other semantic datasets such as Chem2Bio2RDF (Chen et al., 2010). Moreover, we plan to experiment with different pooling operations in both article and query embeddings and present a full parameter sensitivity and ablation studies.

Its worth mentioning that to experiment on a huge KG of billions of nodes, we need a parallel large-scale heterogeneous embedding algorithm that could take in billions of nodes that would presumably be extracted from the whole PubMed corpus. Those models though exist and some of them are used in the industry they can be impractical in research. Most graph embedding algorithms work on a very limited amount of data. Our sample corpus here provides some evidence that this framework is effective and provides better search results than traditional methods opening the door to building a full-scale system. Finally even though this framework here does not address query intention particularly. Yet it considers semantics and relations between terms in the ranking. Semantics could be seen as a step toward future systems that consider query intentions.

## 6. Conclusion

In this article, we presented a proof-of-concept method to build a semantic search engine for the biomedical literature indexed in PubMed named MedGraph. We showed that our method is superior to more traditional approaches in relevance ranking and provided evidence that semantic methods in information retrieval are more needed. Furthermore, we performed a complete evaluation using various metrics on our approach using PubMed's BestMatch as a ground truth. We also presented an innovative way of converting relational databases to KGs. In the future, we hope to expand this work and provide a fully working model and system accessible by researchers to provide better ways to discover knowledge and advance science.

## Data availability statement

Publicly available datasets were analyzed in this study. This data can be found at: The University of Texas at Austin, Domain Informational Vocabulary Extraction (DIVE), PubMed Knowledge Graph Datasets, http://er.tacc.utexas.edu/datasets/ped.

## Author contributions

IE created this framework and wrote the article under the guidance of Dr. Elizabeth Pierce at The University of Arkansas at Little Rock.

## Conflict of interest

The author declares that the research was conducted in the absence of any commercial or financial relationships that could be construed as a potential conflict of interest.

## Publisher's note

All claims expressed in this article are solely those of the authors and do not necessarily represent those of their affiliated organizations, or those of the publisher, the editors and the reviewers. Any product that may be evaluated in this article, or claim that may be made by its manufacturer, is not guaranteed or endorsed by the publisher.

## Footnotes

^1^ http://www.nlm.nih.gov/pubs/factsheets/medline.html

^2^ https://pubmed.ncbi.nlm.nih.gov/

^3^ https://www.nih.gov/

^4^ https://pubmed.ncbi.nlm.nih.gov/

^5^ https://www.nlm.nih.gov/mesh/meshhome.html

^6^ https://www.ncbi.nlm.nih.gov/pmc/
