# Supplementary material for: MedGraph: A semantic biomedical information retrieval framework using knowledge graph embedding for PubMed
Source: Front Big Data. 2022 Oct 19;5:965619. doi: 10.3389/fdata.2022.965619 (PMC9627348; doi:10.3389/fdata.2022.965619)
Supplement: Supplementary file 1 [file Data_Sheet_1.pdf]

Supplementary Material

1 MORE ON KNOWLEDGE GRAPHS AND THEIR EMBEDDING TECHNIQUES

1.1 Knowledge Graphs

The term **Knowledge Graph** emerged to describe a new Google search technology in 2012 Paulheim (2017). The term has expanded to describe any form of a knowledge base represented using a graph structure Ji et al. (2020). Knowledge graphs are data models used to represent structured and unstructured knowledge in triples [Mount Fuji, isLocatedIn, Japan] Ji et al. (2020). The triples represent a semantic relationship between two objects following language semantics written in first-order logic such as [subject, verb, object]. Hence, a knowledge graph represents interrelated and semantically connected descriptions of real-world entities and relationships Ji et al. (2020). The definitions of a knowledge graph vary widely Li et al. (2020). Here I assume the definition described in Paulheim (2017). In Paulheim (2017), the authors identified a set of properties that pertains to a knowledge graph that is:

- They describe real-world phenomena in the form of entities and relationships.
- The entities and relationships are represented in a graph structure using vertices and edges.
- The graph structure containing the knowledge graph can be described mathematically and represented computationally using graph theory norms.
- The entities' metadata and relationships do not necessarily adhere to a schema or an ontology.
- Entities of different types could be linked with multiple edges.
- It could cover multiple domains of knowledge.

Knowledge graph construction can vary from manual to automatic curation by extracting entities and relations from unstructured text Paulheim (2017). Knowledge graphs can be represented using the property graph model Angles (2018). Knowledge graphs can also be represented using the Resources Descriptors Framework Lassila et al. (1998), a semantic representation language with a graph data model defined by the World Wide Web Consortium. A relationship is defined between two connected entities or nodes, such as our previous example [Mount Fuji, isLocatedIn, Japan]. In that example [Mount Fuji] and [Japan] are two unique nodes, vertices or entities in a graph, while [isLocatedIn] is the relationship or edge label connecting them. The RDF standard requires storing node names using a Unique Resource Identifier (URI) format. Knowledge graphs can be constructed and curated under two assumptions: open-world or closed-world assumptions Cai et al. (2018). In the open-world assumption, information outside the knowledge graph is assumed to be unknown, and the knowledge graph itself is considered incomplete, such as DBpedia Bizer et al. (2009). In a closed world assumption, information outside the knowledge graph is false. The data model in the closed-world assumption can be defined in a schema or ontology described using the RDFS language or the Resource Descriptor Framework Schema Lassila et al. (1998). A schema is a concept map or ontology describing entities in the knowledge graph and their relationships.

Like relational databases, knowledge graphs are graph databases queried using query languages Hogan et al. (2021). For example, SPARQL [1] and Cypher [2] are two popular graph query languages like the Structured Query Language (SQL). SPARQL was developed as part of the semantic web technology stack

[1] https://www.w3.org/TR/sparql11-query/

[2] https://neo4j.com/developer/cypher/

to query RDF triple stores and knowledge graphs, while Cypher is a property graph query language Hogan et al. (2021). However, due to the unconventional structure of the graph model and the fact that knowledge graphs can be multigraphs with attributes and edges that could be weighted or labeled, graph query languages are difficult and impractical for the complex analysis of knowledge graphs. Those limitations have led to the emergence of knowledge graph mining methods and algorithms especially embedding models borrowing from graph theory, complex networks, machine learning, and deep learning to adapt approaches developed in those fields to mine knowledge graphs more flexibly and extract deeper insights Wang et al. (2017).

1.2 Knowledge Graph Embedding and Similarity

Knowledge graph embedding models automatically extract latent feature vectors from data without relying on stochastic and heuristic metrics and measures Bengio et al. (2013). All representation learning algorithms on graphs ultimately produce node embedding vectors in low-dimensional vector space Hamilton et al. (2017). The minimal constraint on the learned representations is that they preserve the graph's structure in the Euclidean vector space Hamilton et al. (2017). The node embedding vectors learned can be passed to downstream machine learning models for classification, regression, or clustering. For example, cosine distance could be applied to the learned vectors to rank the nodes compared to a query node. Alternatively, they can be used directly in the learning process in a semi-supervised end-to-end fashion as in the Graph Neural Network (GNN) model Wu et al. (2020). They can also be transductive, as in learned from the structure of the graph itself Perozzi et al. (2014) Tang et al. (2015) Grover and Leskovec (2016), or distance based by forcing a scoring function to evaluate the plausibility of the triples in the knowledge graph Lin et al. (2015).

The Skip-gram model described in Mikolov et al. (2013b) is an unsupervised language model aiming at learning discrete vectors of unique words given a corpus of text. The Skip-gram model has been adapted to learn vectors for individual nodes on a corpus of sampled nodes from the graph in unsupervised network and graph embedding algorithms. In DeepWalk Perozzi et al. (2014), the authors first proposed using the Skip-gram model on a corpus of sampled nodes from a given graph using a random walk strategy to sample the graph. Since it was observed that the distribution of words in any corpus of text followed Zipf's law, it was also observed that the frequency distribution of node degrees in a graph follows Zipf's law Perozzi et al. (2014). The random walk algorithm aims at sampling chains of nodes from the graph controlled by the walk length. Sampled chains are then treated as sentences in a text corpus fed to a Skip-gram variant of Word2vec Mikolov et al. (2013a).

2 ILLUSTRATIVE EXAMPLE SUPPLEMENTAL TO FIGURE 1

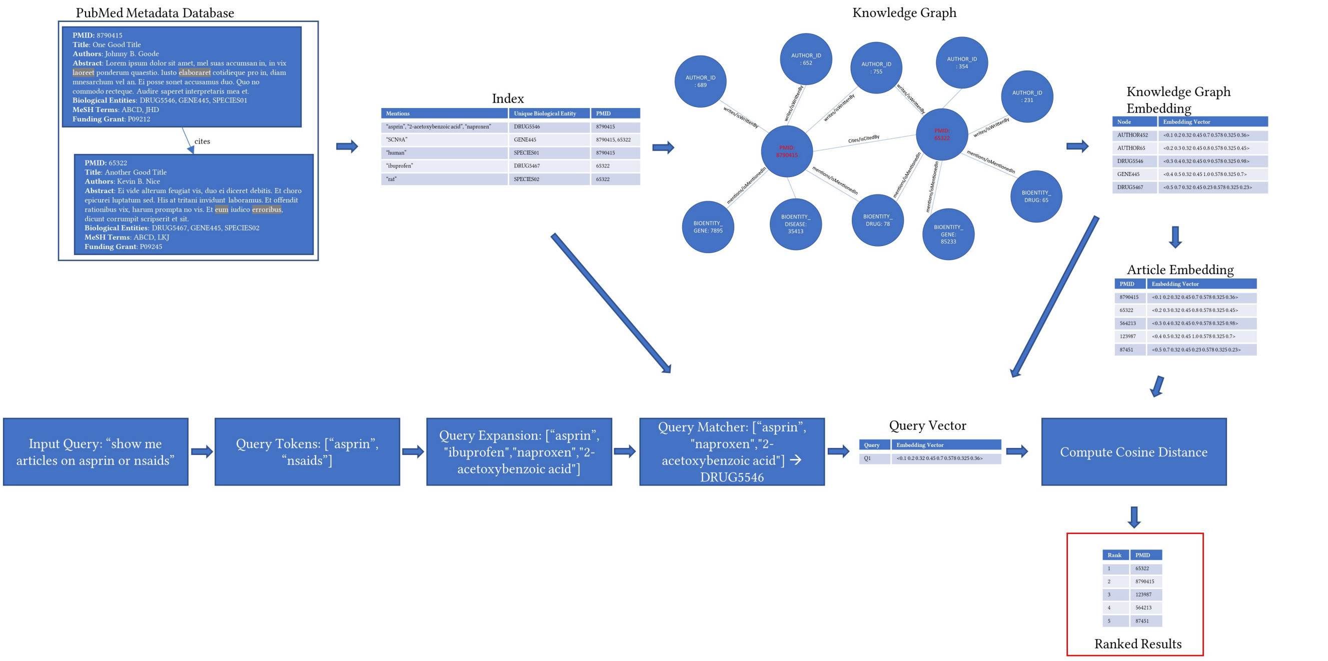

Figure S1. The figure shows in an illustrative way the content of the pipeline in figure 1. The first input database contains abstracts and other metadata for each article. Second an index is created from the recognized entities from abstracts. Then a knowledge graph is created based on the co-occurrence of the unique entities in the articles and the citation network. Next the integrated knowledge graph is embedded. Then articles embeddings are created using mean pooling. On the query size at the bottom of the image first the input query is tokenized then expanded then matched with the index. Then a query vector is created. Following a cosine distance is computed between the query vector and the article vectors producing the ranked articles.

REFERENCES

Angles, R. (2018). The Property Graph Database Model. In *AMW*. 1–10

Bengio, Y., Courville, A., and Vincent, P. (2013). Representation learning: A review and new perspectives. *IEEE transactions on pattern analysis and machine intelligence* 35, 1798–1828. Publisher: IEEE

Bizer, C., Lehmann, J., Kobilarov, G., Auer, S., Becker, C., Cyganiak, R., et al. (2009). Dbpedia-a crystallization point for the web of data. *Journal of web semantics* 7, 154–165. Publisher: Elsevier

Cai, H., Zheng, V. W., and Chang, K. C.-C. (2018). A comprehensive survey of graph embedding: Problems, techniques, and applications. *IEEE Transactions on Knowledge and Data Engineering* 30, 1616–1637. Publisher: IEEE

Grover, A. and Leskovec, J. (2016). node2vec: Scalable feature learning for networks. In *Proceedings of the 22nd ACM SIGKDD international conference on Knowledge discovery and data mining*. 855–864

Hamilton, W. L., Ying, R., and Leskovec, J. (2017). Representation learning on graphs: Methods and applications. *arXiv preprint arXiv:1709.05584*

Hogan, A., Blomqvist, E., Cochez, M., d'Amato, C., Melo, G. d., Gutierrez, C., et al. (2021). Knowledge graphs. *Synthesis Lectures on Data, Semantics, and Knowledge* 12, 1–257. Publisher: Morgan & Claypool Publishers

Ji, Y., Yin, M., Yang, H., Zhou, J., Zheng, V. W., Shi, C., et al. (2020). Accelerating Large-Scale Heterogeneous Interaction Graph Embedding Learning via Importance Sampling. *ACM Transactions on Knowledge Discovery from Data (TKDD)* 15, 1–23. Publisher: ACM New York, NY, USA

Lassila, O., Swick, R. R., et al. (1998). Resource description framework (rdf) model and syntax specification. *World Wide and Web Consortium*

Li, Y., Cai, W., Li, Y., and Du, X. (2020). Key node ranking in complex networks: A novel entropy and mutual information-based approach. *Entropy* 22, 52. Publisher: Multidisciplinary Digital Publishing Institute

Lin, Y., Liu, Z., Sun, M., Liu, Y., and Zhu, X. (2015). Learning entity and relation embeddings for knowledge graph completion. In *Twenty-ninth AAAI conference on artificial intelligence*. 2181–2187

Mikolov, T., Chen, K., Corrado, G., and Dean, J. (2013a). Efficient estimation of word representations in vector space. *arXiv preprint arXiv:1301.3781*

Mikolov, T., Sutskever, I., Chen, K., Corrado, G. S., and Dean, J. (2013b). Distributed representations of words and phrases and their compositionality. In *Advances in neural information processing systems*. 3111–3119

Paulheim, H. (2017). Knowledge graph refinement: A survey of approaches and evaluation methods. *Semantic web* 8, 489–508. Publisher: IOS Press

Perozzi, B., Al-Rfou, R., and Skiena, S. (2014). Deepwalk: Online learning of social representations. In *Proceedings of the 20th ACM SIGKDD international conference on Knowledge discovery and data mining*. 701–710

Tang, J., Qu, M., Wang, M., Zhang, M., Yan, J., and Mei, Q. (2015). Line: Large-scale information network embedding. In *Proceedings of the 24th international conference on world wide web*. 1067–1077

Wang, Q., Mao, Z., Wang, B., and Guo, L. (2017). Knowledge graph embedding: A survey of approaches and applications. *IEEE Transactions on Knowledge and Data Engineering* 29, 2724–2743. Publisher: IEEE

Wu, Z., Pan, S., Chen, F., Long, G., Zhang, C., and Philip, S. Y. (2020). A comprehensive survey on graph neural networks. *IEEE transactions on neural networks and learning systems* 32, 4–24
